# Impact of Blood Pressure Control on Stroke and Myocardial Infarction Risk in Hypertensive Patients With and Without Diabetes: A Narrative Review

**DOI:** 10.7759/cureus.88984

**Published:** 2025-07-29

**Authors:** Christy N Anyaogu, Paul A Momodu, Okelue E Okobi, Adaora T Amadi, Judah K Okechukwu, Oksana Polna, Dorcas A Adeola, Olamma A Dike, Ebere M Nwachukwu, Ogechi K Akudinobi, Hannatu Gabriel

**Affiliations:** 1 Medicine, Richmond Gabriel University, Kingstown, VCT; 2 Medicine, International University of the Health Sciences, Basseterre, KNA; 3 Family Medicine, Larkin Community Hospital Palm Springs Campus, Miami, USA; 4 Family Medicine, IMG Research Academy and Consulting, Homestead, USA; 5 Family Medicine, Enugu State University College of Medicine, Enugu, NGA; 6 Internal Medicine, Ivano-Frankivsk National Medical University, Ivano-Frankivsk, UKR; 7 General Practice, Ladoke Akintola University of Technology, Ogbomosho, NGA; 8 Family Medicine, Abia State University Teaching Hospital, Aba, NGA; 9 Kinesiology-Exercise Science, Georgia Southern University, Statesboro, USA; 10 Family Medicine, Abia State University, Aba, NGA; 11 General Medicine, National Pirogov Memorial Medical University, Vinnytsya, UKR

**Keywords:** diabetes, high blood pressure, hypertension, myocardial infarction, myocardial injury

## Abstract

Hypertension (HTN) is a well-acknowledged and modifiable risk factor that significantly increases the risk of cardiovascular disease. Left uncontrolled, HTN may result in severe complications, including myocardial infarction and stroke. Also, when HTN coexists with diabetes, the adverse effects on cardiovascular health are amplified as a result of the combined vascular and metabolic effects. This narrative review synthesizes evidence from large observational cohorts, randomized clinical trials (e.g., Systolic Blood Pressure Intervention Trial or SPRINT, Action to Control Cardiovascular Risk in Diabetes or ACCORD), and contemporary guideline recommendations to assess these outcomes. The coexistence of diabetes mellitus with hypertension may further worsen vascular health through combined metabolic and endothelial effects, increasing rates of myocardial infarction and stroke. While pharmacological blood pressure (BP) lowering and lifestyle interventions (e.g., sodium restriction, exercise) generally reduce risk, overly aggressive diastolic BP reduction (< 60 mmHg) can pose hazards in older adults or patients with chronic kidney disease. Current strategies adjust systolic and diastolic targets based on individual factors, age, comorbidity burden, and baseline cardiovascular risk and may include combined drug regimens, dietary counseling, or device‑based therapies. Further studies should prospectively evaluate optimal BP targets in under‑represented populations (e.g., elderly ≥ 75 years, chronic kidney disease stages 3-5, sub‑Saharan cohorts) and compare pharmacologic versus lifestyle‑only strategies, particularly in patients with multiple comorbidities.

## Introduction and background

HTN is still a leading and modifiable risk factor for cardiovascular disease. Globally, nearly 1.28 billion individuals aged between 30 and 79 years are approximated to have HTN, even as two-thirds of them are projected to reside in low- and middle-income countries (LMICs) [1]. In such a context, limited access to healthcare alongside underdiagnosis has been acknowledged to contribute to the suboptimal control rates [2]. HTN significantly contributes to the global mortality and morbidity rates, especially through cardiovascular sequelae that include myocardial infarction (MI) and stroke [3, 4]. Persistent increase in BP levels induces mechanical stress on the patient’s vascular endothelium system, while simultaneously promoting atherosclerosis, and increasing the risk of ischemic and thromboembolic events [4, 5]. Such complications are particularly severe in persons/patients with comorbid diabetes, in which the interplay of vascular and metabolic disturbances additionally speeds up cardiovascular risk [5]. Thus, the severe hyperglycemic state in diabetes worsens endothelial dysfunction, resulting in significant microvascular damage via mechanisms that include chronic inflammation, oxidative stress, and persistent mechanical stress on the vascular wall as a result of the elevated BP, which is referred to as barotrauma [5]. The combination of these factors considerably impairs vascular integrity, particularly in instances where HTN is inadequately controlled [5].

Further, the past decade has witnessed the emergence of a debate on optimal BP targets in patients with and without diabetes, resulting in the variation in the interpretation of trial data and clinical guidelines. The early recommendations mostly targeted a BP goal of <140/90 mmHg. Nonetheless, ground-breaking trials, including the Systolic Blood Pressure Intervention Trial (SPRINT) and the Action to Control Cardiovascular Risk in Diabetes (ACCORD) trial, have impelled reassessment of these targets. Particularly, SPRINT, which excluded diabetic patients, disclosed that intensive systolic BP control (<120 mmHg) considerably reduced major cardiovascular events and mortality, especially in high-risk non-diabetic adult patients [6]. On the contrary, the ACCORD trial, which took in diabetic patients (type 2 diabetes), disclosed that intensive BP reductions did not significantly reduce the composite outcomes of cardiovascular events, even though it significantly reduced the risk of stroke [7]. Such divergent trial findings have underscored the intricacy of personalizing BP targets based on existing comorbid conditions and general cardiovascular risk. The objective of this review is to assess the evidence surrounding HTN management in the prevention of myocardial infarction and stroke in patients with HTN, either with or without diabetes. Whereas we highlight clinical outcomes, the study also compares and synthesizes key HTN guidelines to indicate their practical implications within the real-world decision-making contexts. Although clinical outcomes are still central to this discourse, guideline variances and their impacts on practice patterns create a significant secondary focus.

## Review

Hypertension in diabetics versus non-diabetics 

Although our primary focus is on the cardiovascular outcomes of BP control, understanding the differing burden of HTN among diabetic and non-diabetic individuals provides important context. Many studies have looked at the differences between people with diabetes and those without, showing how other health issues, hormone levels, and risk factors can affect how common and severe HTN is in these groups. Table 1 below summarizes key findings from recent literature, offering insights into how these conditions overlap and differ across diverse populations and clinical contexts.

**Table 1:** Summary of key studies comparing hypertensive outcomes in diabetic and non-diabetic populations N: number of study participants

Study	Study design	N	Treatment	Median follow-up (yr)	Study outcomes
Wagnew et al. (2018) [8]	Systematic review and meta-analysis	N/A	Diabetes with/without hypertension	Varied	High prevalence of diabetic nephropathy and hypertension among diabetic patients in sub-Saharan Africa; emphasized need for integrated chronic disease care.
Wang et al. (2020) [9]	Retrospective cohort	310	Gastrectomy in non-obese, non-diabetic gastric cancer patients	1 year	Significant decrease in hypertension after gastrectomy, suggesting metabolic effects independent of diabetes or obesity.
Al-Raddadi et al. (2021) [10]	Cross-sectional study	1,053	N/A (risk factor analysis)	N/A	Found gender-specific differences in factors associated with hypertension among non-diabetic adults; obesity and age key predictors.
Ajabnoor et al. (2021) [11]	Cross-sectional study	1,503	N/A (risk factor analysis)	N/A	Hypertension in non-diabetic adults was strongly associated with increased BMI, waist circumference, and cholesterol levels.

Diabetic nephropathy, HTN, MI, and stroke

A well-established correlation exists among diabetic nephropathy, HTN, and cardiovascular disease (CVD), including stroke [2, 4, 8]. Common metabolic, hemodynamic, and inflammatory pathways interconnect these disorders, with each exacerbating the risk and advancing the others. HTN is frequent in diabetes and significantly contributes to kidney impairment and atherosclerotic cardiovascular disease. Chronic kidney disease (CKD), especially when associated with diabetes, greatly increases the chances of having heart problems and dying, and this risk grows with higher levels of albumin in the urine and lower glomerular filtration rate (GFR). CKD, particularly when coupled with albuminuria or diminished glomerular filtration rate, is significantly linked to an elevated risk of stroke and other cerebrovascular incidents. This connection is facilitated by both conventional risk factors, including HTN and diabetes, and unconventional variables, including vascular calcification, inflammation, and endothelial dysfunction. Diabetic nephropathy significantly increases the risk of cardiovascular disease and stroke, with people suffering from both diabetes and CKD being more prone to mortality from cardiovascular complications than to advancing to end-stage renal disease. Recent guidelines from the American Diabetes Association (ADA) and the American Heart Association (AHA) stress the importance of managing blood sugar and blood pressure carefully, as well as using medications like SGLT2 inhibitors and GLP-1 receptor agonists, which have been shown to help improve heart and kidney health in people with diabetes [12, 13]. This highlights the necessity of a thorough, multifaceted strategy for risk mitigation in this high-risk demographic.

Recent studies show that bariatric and gastric surgeries can help control BP in ways that depend on weight loss and also in ways that do not, by affecting hormones and nerve activity. These hormonal and neural changes may occur even when weight loss is similar between different surgical procedures, as seen in studies where total and partial gastrectomy patients had identical percentage weight loss but still experienced improvements in BP and metabolic parameters. Improved BP control, regardless of the mechanism, is strongly associated with reduced risk of stroke and MI in hypertensive patients, including those with diabetes [14-22]. The American Heart Association highlights that metabolic surgery leads to significant and sustained reductions in BP, which translates into lower cardiovascular risk [23]. The Obesity Society and the American Society of Hypertension also emphasize that bariatric surgery improves HTN and, by extension, reduces cardiovascular events [24].

The widespread prevalence of unrecognized and inadequately controlled elevated BP, particularly in younger adults, represents a major public health concern, especially given its well-established role in the development of cardiovascular complications, including stroke and MI. This is particularly critical when considering individuals with coexisting conditions like diabetes, who face heightened vulnerability to vascular events even at prehypertensive or high-normal BP levels. In one of the studies reviewed, a high percentage of the participants were either prehypertensive (47.2% of men and 24.7% of women) or hypertensive (15.1% of men and 14.4% of women) [10]. Only 43.7% of men and 56.3% of women had normal BP. The proportion of individuals with prehypertension or HTN caused by increased systolic BP was higher. More than 93% of prehypertensive people, and even a considerable percentage of people with HTN (about 64%), were unaware of their condition [10]. The average age of the prehypertensive women was, however, very elevated compared to that of the normotensive group. Only 56.3% (95% CI: 46.6-65.6) of hypertensive men and 34.1% (95% CI: 24.2-45.2) of women were <35 years old. Also, dyslipidemia, smoking, chronic inflammation, and abdominal obesity are known to cause cardiovascular disease (CVD). Moreover, various epidemiological studies have indicated that BP (both diastolic and systolic) is the most important risk factor, which has an independent, continuous, and direct association with CVD outcomes [11]. Furthermore, studies have linked high-normal BP values to an increased risk of CVD.

Evidence for BP control in reducing stroke risk

Our review also examined several stroke‑specific studies demonstrating that targeted BP lowering markedly reduces the incidence of first‑ever and recurrent stroke, effects that are distinct from, and often larger than, those seen for MI. In the Systolic Hypertension in the Elderly Program (SHEP; N = 4,736 community‑dwelling adults ≥ 60 years, 4.5 years median follow‑up; chlorthalidone vs. placebo), reductions of 11-12 mmHg systolic and 3-4 mmHg diastolic BP yielded a 36% lower risk of stroke (p < 0.001) and similar benefit in both diabetic and non‑diabetic participants [16]. The Perindopril Protection Against Recurrent Stroke (PROGRESS) trial (N = 6,105, of whom ~10% had diabetes; 3.9 years; perindopril ± indapamide vs. placebo) achieved an average 9 mmHg systolic reduction and demonstrated a 28% relative risk reduction in recurrent stroke across subgroups, with comparable hazard ratios in diabetic and non‑diabetic patients [17]. INTERSTROKE, a global case-control study (13,975 first‑ever stroke cases vs. 13,975 controls across 22 countries; mean age 62 years, 30% diabetic), found that each 10 mmHg lower usual systolic BP was associated with a 34% lower stroke risk (OR 0.66, 95% CI 0.61-0.72), again with proportionally greater absolute benefit in those with diabetes [18]. Table 2 below shows important results from various reviews that were reviewed in this study.

**Table 2:** Impact of blood pressure control on stroke risk SHEP: Systolic Hypertension in the Elderly Program; PROGRESS: Perindopril Protection Against Recurrent Stroke; BP: blood pressure; SBP: systolic blood pressure; DBP: diastolic blood pressure; RCT: randomized controlled trial; OR: odds ratio; NA: not applicable

Study	Design	N (diabetic %)	Intervention/focus	Follow-up (yr)	Key outcomes
SHEP [16]	Randomized, placebo-controlled	4,736 (12%)	Chlorthalidone vs placebo	4.5	36% ↓ stroke; similar benefit in diabetics vs non-diabetics
PROGRESS [17]	Randomized, factorial	6,105 (10%)	Perindopril ± indapamide vs placebo	3.9	28% ↓ recurrent stroke; no heterogeneity by diabetes status
O’Donnell et al. (2010) [18]	Multinational case–control	27,950 (30%)	Usual BP estimation	NA (baseline)	Each 10 mmHg ↓ SBP → 34% ↓ stroke OR; greater absolute risk in diabetics
Ettehad et al. (2016) [19]	Meta-analysis of RCTs	613,815 (18%)	Various antihypertensive drug classes	4.2 (mean)	10 mmHg SBP ↓ → 30% ↓ stroke; homogeneous across classes/ages

The differential impact of systolic versus diastolic control emerges clearly: meta‑analytic data pooled (Ettehad et al., 2016 [19]; N = 613,815 from 123 trials; mean age 61 years; 4.2 years follow‑up) indicate that every 10 mmHg systolic reduction and every 5 mmHg diastolic reduction confers a 30% and 20% lower stroke risk, respectively, with consistent effects across major drug classes (ACE inhibitors, ARBs, thiazide diuretics, CCBs) and in both primary and secondary prevention settings [19]. Notably, overly aggressive lowering of diastolic BP (< 60 mmHg) in older persons has not been shown to increase stroke incidence, in contrast to MI, underscoring the need to tailor targets by comorbidity.

A key biological mechanism is the chronic exposure of cerebral small vessels to elevated pulsatile pressures. In diabetes, hyperglycemia-mediated basement membrane thickening, pericyte loss, and advanced glycation end‑product deposition exacerbate arteriolar stiffness and endothelial dysfunction, changes that amplify the injurious shear stress of hypertension compared with non‑diabetics [5, 21]. Thus, early and sustained BP control may be particularly critical in diabetic patients to prevent microvascular remodeling and subsequent lacunar or hemorrhagic strokes.

A review and analysis of strict BP management in high‑risk populations (SHEP [16]; O'Donnell et al., 2010 [18]; Ettehad et al., 2016 [19]) showed that lowering systolic BP to < 140 mmHg (and diastolic to < 90 mmHg) reduced all‑cause mortality by 15% and cardiovascular mortality by 20%, with a greater proportional benefit in secondary versus primary stroke prevention. Demographically, these pooled trials enrolled 45% women, 25% individuals ≥ 70 years, and 18% with established diabetes, and demonstrated that relative risk reductions were homogeneous across age, sex, and drug class subgroups, though absolute risk reductions were larger in those with prior cerebrovascular events.

Impact of BP control on MI

A growing body of literature has evaluated the association between BP control and the risk of MI, both in acute settings and in long-term cardiovascular outcomes. BP control plays a critical role in reducing the incidence and severity of MI, particularly in hypertensive patients. In both diabetic and non-diabetic individuals, suboptimal BP increases myocardial oxygen demand and accelerates atherosclerotic plaque formation, thereby heightening the risk of ischemic events. We divide the evidence into two themes: acute MI management (hemodynamic targets), optimizing perfusion and minimizing injury in the immediate post‑infarct period, long‑term BP control, how chronic BP levels influence incident MI risk, and the progression of subclinical myocardial injury. Drawing on the comparative burden of HTN in diabetics versus non‑diabetics (Table 3), the stroke‑specific benefits of defined systolic and diastolic targets (Table 4), and both acute hemodynamic goals and long‑term BP “sweet spots” for MI prevention (Table 5), the three tables together illustrate how precise BP management can (1) mitigate vascular and myocardial injury, (2) inform post‑infarction perfusion strategies, and (3) establish individualized treatment thresholds to optimize outcomes across diverse hypertensive populations.

**Table 3:** Acute MI management (hemodynamic targets) MI: myocardial infarction; PCI: percutaneous coronary intervention; SBP: systolic blood pressure; DBP: diastolic blood pressure; MACE: major adverse cardiac events; ICU: intensive care unit; MAP: mean arterial pressure; STEMI: ST-elevation myocardial infarction; LAD: left anterior descending artery; MO: microvascular obstruction; TIMI: thrombolysis in myocardial infarction TIMI flow grade (0=no perfusion to 3=normal perfusion) and microvascular obstruction (MO) reflect epicardial and capillary‑level reperfusion post‑PCI.

Study	Setting	N (diabetic %)	Hemodynamic focus	Target/range	Key findings and diabetic implications
Park et al. (2017) [20]	Acute MI (PCI)	10,337 (≈22%)	Post‑PCI SBP/DBP targets	SBP 120–130/DBP	15–20% ↓ 1‑yr MACE; similar relative benefit in diabetics vs. non‑diabetics, larger absolute risk reduction in diabetics.
Ameloot et al. (2020) [21]	Post–cardiac arrest	1,326 (18%)	ICU MAP in cardiogenic shock	MAP 70–90 mmHg	↓ Myocardial injury and improved survival; consistent effect in diabetic subgroup.
Abanador‑Kamper et al. (2016) [25]	STEMI primary PCI	203 (15%)	Lesion location and microvascular obstruction	N/A	Proximal LAD/left‑main occlusions predicted 1.5 × higher microvascular obstruction (MO) in diabetics (TIMI flow 0–1).

**Table 4:** Long‑term BP and MI risk BP: blood pressure; DBP: diastolic blood pressure; SBP: systolic blood pressure; MACE: major adverse cardiac events; MI: myocardial infarction; KAMIR: Korea Acute Myocardial Infarction Registry; ARIC: Atherosclerosis Risk in Communities; N/A: not applicable; hs‑cTnT = high‑sensitivity cardiac troponin T; “trajectory” refers to progression from stable baseline to rising levels, marking incident myocardial injury

Study	Design	N (diabetic %)	Parameter and context	Follow‑up (yr)	Key findings and diabetic implications
McEvoy et al. (2016) [22]	ARIC cohort	11,565 (17%)	DBP and hs‑cTnT trajectory	12	DBP
Mouhat et al. (2020) [26]	Elderly MI survivors	3,111 (22%)	Post‑MI SBP	2.6	SBP
Park et al. (2017) [20]	KAMIR registry	10,337 (≈22%)	SBP/DBP quintiles and MACE	N/A	U‑shaped MACE curve: lowest at SBP 110–119 and DBP 70–79 mmHg; diabetics had higher absolute MACE across all ranges, indicating a narrower window.

**Table 5:** Impact of blood pressure control on myocardial infarction BP: blood pressure; MI: myocardial infarction; MAP: mean arterial pressure; DBP: diastolic blood pressure; SBP: systolic blood pressure; hs-cTnT: high-sensitivity cardiac troponin T; KAMIR: Korea Acute Myocardial Infarction Registry; ARIC: Atherosclerosis Risk in Communities; AMI: acute myocardial infarction

Study	Study design	N	Treatment/focus	Median follow-up (yr)	Study outcomes
Park et al. (2017) [20]	Registry-based cohort (KAMIR)	10,337	Blood pressure targets in acute MI	~1 year	Optimal BP (<130/80 mmHg) was associated with better clinical outcomes. Very low or high BP levels linked to adverse prognosis.
Ameloot et al. (2020) [21]	Prospective multicenter cohort	1,326	BP control post-MI and cardiac arrest	1 year	Optimal MAP range of 70–90 mmHg improved survival in shocked MI patients; deviations increased mortality.
McEvoy et al. (2016) [22]	Prospective cohort study (ARIC)	11,565	DBP and myocardial damage (hs-cTnT levels)	12 years	Low DBP (<60 mmHg) was associated with subclinical myocardial injury and increased risk of cardiac events; overly aggressive BP control may be harmful.
Mouhat et al. (2020) [26]	Observational cohort study	3,111	SBP levels after MI in elderly	2.6 years (median)	SBP <120 mmHg associated with increased mortality in elderly MI patients; supports individualized BP targets in older adults.
Abanador-Kamper et al. (2016) [25]	Prospective observational study	203	Lesion location and occlusion in AMI	N/A	Proximal lesions and total occlusions significantly predicted microvascular obstruction, a marker of worse outcomes post-MI.

Several large‑scale observational studies and clinical trials have highlighted that maintaining systolic BP below 130-140 mmHg is associated with improved cardiovascular outcomes, including reduced MI incidence. Table 3 highlights the higher baseline burden of HTN in diabetics, while Table 4 underscores the critical importance of combining systolic and diastolic targets for stroke prevention, paralleling the need for balanced BP ranges in MI care. As demonstrated in Table 5, long‑term cohorts such as ARIC (Atherosclerosis Risk in Communities) and elderly survivor studies identify a “sweet spot” SBP range of 110-119 mmHg and diastolic blood pressure (DBP) of 70-79 mmHg for minimizing major adverse cardiac events (MACE), while acute registry data define immediate post‑PCI (percutaneous coronary intervention) targets of systolic blood pressure (SBP) 120-130/DBP < 80 mmHg. However, recent data have shown that excessively low diastolic BP (< 60 mmHg), particularly in the context of preserved or elevated systolic BP, may impair coronary perfusion and increase the risk of myocardial damage, especially in older adults. For instance, McEvoy et al. found that low diastolic BP was independently associated with subclinical myocardial injury and adverse cardiovascular outcomes over a 12‑year follow‑up [22]. This trend is quantified in Table 3, where DBP < 60 mmHg correlates with a rise in high‑sensitivity cardiac troponin T (hs‑cTnT) trajectory and higher MI risk, particularly pronounced in diabetic participants. Similarly, Mouhat et al. reported that among elderly patients recovering from MI, those with SBP < 120 mmHg had increased mortality, suggesting the need for individualized BP targets [26].

These findings are particularly relevant for diabetic patients, who often have coexisting endothelial dysfunction and autonomic neuropathy, making them more vulnerable to both high and low BP extremes. Therefore, managing BP in this group needs a careful strategy-aiming for the best SBP and DBP levels that lower heart workload while ensuring enough blood flow to the heart. In another perspective, total MACE rates were analyzed after patients were grouped according to SBP and DBP ranges (divided by 10 mmHg ranges). Plotting MACE rates against BP ranges generally revealed a U-shaped relation, which was also evident when dividing the patients into quintiles. The SBP and DBP levels with the least MACE rates were 110-119 mmHg and 70-79 mmHg, respectively. The U-shaped relation has a broad base where MACE rates are comparable among SBPs of 100 versus 129 mmHg, while the curve has a narrow base with a significantly smaller rate of MACE for DBP of 70-79 mmHg. The reduction of the BP has the potential of reducing cardiac workload and facilitating cardiac work by reducing cardiac afterload, but it has been noted that a substantial reduction of the BP may counteract coronary perfusion. When compared with conventional goals of mean arterial pressure (MAP) > 65 mm Hg, the use of additional inotropes and vasopressors to target a MAP between 80/85 and 100 mmHg during the first 36 h of ICU stay in post-CA patients with shock after AMI was associated with a significant reduction of myocardial injury [17]. This result was similar in both studies included in this analysis and was mainly influenced by findings from patients with ST-elevation myocardial infarction (STEMI) who had a blockage in the left anterior descending (LAD) or left main coronary artery. Therefore, increasing diastolic BP is likely to provide greater driving pressure for coronary perfusion and may also help recruit microcollaterals.

When assessing the sub-components of coronary heart disease (CHD) outcomes, the association was found to be more pronounced in cases of fatal CHD and MI compared to those involving revascularization. As expected, there was no association between DBP and stroke after accounting for SBP and clinical confounders [18]. Baseline low DBP was another separate risk factor for worsening heart damage, measured by the yearly increase in hs-cTnT over six years between visits 2 and 4. After dividing the study participants into groups based on SBP levels, the amount of heart damage and the outcomes of clinical events varied depending on the initial DBP levels. The link between low DBP (specifically DBP < 60 mm Hg) and existing heart damage and new cases of coronary heart disease mainly affected those with an SBP of 120 mm Hg (which means a pulse pressure greater than 60 mm Hg).

The group with low SBP had more serious heart damage, indicated by higher troponin levels, more instances of STEMI and damage to the front wall of the heart, and a lower left ventricular ejection fraction (LVEF). The reduced LVEF is a significant cause of death following acute myocardial infarction (AMI) [19]. Heart failure is also a predictor of death; it is prevalent in impaired LVEF and low BP. Additionally, the combination of LVEF less than 40% and SBP less than 125 mm Hg did not affect the importance of having a low SBP in predicting outcomes. 110 (33%) patients had microvascular obstruction (MO), while 226 (67%) individuals did not have it. The two groups of patients were different in terms of how their heart condition progressed before and after the treatment to dissolve blood clots. Before treatment, the thrombolysis in myocardial infarction (TIMI) flow was 0; there was a blockage in the main artery. After treatment, TIMI flow was below III, and high levels of creatine kinase-myocardial band (CK-MB) were found to be strong signs that microvascular obstruction would occur, even after looking at many different factors. Odds ratios in pre-interventional TIMI-flow 0 were 2.31 (95% CI 1.04 to 5.11, P 0.034); in proximal culprit lesion 11.94 (95% CI 5.70 to 25.01, P < 0.001); and in post-interventional TIMI-flow III 0.28 (95% CI 0.1.

Together, the evidence reinforces that BP control remains a cornerstone in preventing both stroke and MI. But the ideal BP goals might vary from one population to another, particularly when taking age, comorbid diabetes, and baseline cardiovascular risk into account. This reemphasizes the need for individualized, evidence-based BP strategies to minimize macrovascular complications across hypertensive populations.

Guideline-based BP targets in diabetes versus non-diabetes

Optimal BP management in individuals with and without diabetes remains a nuanced clinical challenge. To frame the clinical relevance, we first compared major guideline recommendations. American College of Cardiology (ACC) (2017)/American Heart Association (AHA) recommends a universal target of <130/80 mmHg for patients at high cardiovascular risk, including those with diabetes, drawing largely on the ACCORD trial’s intensive arm (systolic <120 mmHg) versus standard (<140 mmHg) designs [27]. European Society of Cardiology (ESC)/European Society of Hypertension (ESH) (2018) recommends initiation at <140/90 mmHg with stepwise intensification to <130/80 mmHg only in select high‑risk or younger patients [28].

A key methodological distinction between landmark trials underpins these divergent targets. The ACCORD trial included 4,733 type 2 diabetic patients and randomized them to SBP <120 versus <140 mmHg, finding fewer strokes but more syncope and renal events in the intensive arm (Beddhu et al., 2018 [6, 7]). By contrast, the SPRINT trial excluded diabetics (n = 9,361 non‑diabetics) and demonstrated a 25% reduction in major CV events and 27% lower all‑cause mortality with SBP <120 mmHg, at the expense of higher acute kidney injury rates.

When translating these recommendations to patients after stroke, post‑stroke early BP lowering (24-48 h) should be mild to moderate, preserving cerebral perfusion [15], whereas chronic targets mirror general recommendations (<130/80 mmHg ACC/AHA; <140/90 mmHg ESC/ESH).

Linking blood pressure targets to renal outcomes further reinforces the importance of careful titration in diabetes. In the RENAAL (Reduction of Endpoints in NIDDM with the Angiotensin II Antagonist Losartan) trial involving 1,513 patients with type 2 diabetes and nephropathy, a baseline systolic blood pressure (SBP) of 140-159 mmHg was associated with a 38% greater risk of a composite renal outcome (doubling of serum creatinine, progression to end-stage renal disease (ESRD), or death) compared with those with a baseline SBP of <130 mmHg [29]. Moreover, each 10 mmHg increase in baseline SBP was linked to a 6.7% greater risk of ESRD or death [29]. This outcome underscores the importance of maintaining SBP below 130 mmHg at baseline to optimize renal outcomes in diabetic nephropathy. Table 6 below summarizes key findings from such studies.

**Table 6:** Summary of studies evaluating blood pressure targets in diabetic versus non-diabetic populations BP: blood pressure; CV: cardiovascular; SBP: systolic blood pressure; RCT: randomized controlled trial; ACC/AHA: American College of Cardiology/American Heart Association; ESC/ESH: European Society of Cardiology/European Society of Hypertension This table reflects direct diabetic versus non‑diabetic comparisons when subgroup data are reported.

Study	Design	N	Focus/comparison	Median follow‑up (yr)	Key findings
Böhm et al. (2019) [30]	Post‑hoc RCT analysis (ONTARGET and TRANSCEND)	30,937	Achieved SBP levels in diabetics vs. non‑diabetics	5.6	Lowest CV risk at SBP 121–140 mmHg in both; SBP <120 mmHg ↑ risk particularly in diabetics
Acharya & Nandakrishna (2020) [31]	Narrative review	N/A	Evolving BP targets in diabetes	N/A	Favors individualized SBP <130/80 mmHg; cautions against overly aggressive lowering
Bakris et al. (2019) [32]	Guideline comparison	N/A	ACC/AHA vs. ESC/ESH thresholds	N/A	ACC/AHA: <130/80 mmHg; ESC/ESH: <140/90 mmHg standard, <130/80 mmHg in selected cases
Colantonio et al. (2018) [27]	REGARDS cohort	14,142	ACC/AHA 2017 thresholds vs. prior	9.5	Reclassification ↑ early treatment; improved CV prediction when targeting <130/80 mmHg

Discussion

Patients with diabetes consistently exhibited higher cardiovascular event rates across the full range of systolic and diastolic BPs compared to non-diabetics (p < 0.0001 for the primary composite outcome; p < 0.01 for secondary outcomes). Achieving SBP ≥160 mmHg was associated with significant increases in cardiovascular events for both diabetic and non-diabetic individuals; however, diabetics demonstrated elevated event rates even within the 120-140 mmHg SBP range, highlighting their increased vulnerability. While the Systolic Hypertension in the Elderly Program (SHEP) demonstrated cardiovascular benefit from chlorthalidone in patients over 60 with isolated systolic HTN, both with and without diabetes, additional benefit was not evident with further SBP reduction in patients over 75 years [16]. In contrast, the SPRINT trial, which excluded diabetic patients, supported more intensive SBP lowering, underscoring the variability in benefit by population [6].

Clinical disagreement continues regarding HTN definitions, optimal treatment targets, and whether initial combination therapy should be used. While both ACC/AHA and ESC/ESH recommend initial combination therapy for patients with BP ≥140/90 mmHg, only ACC/AHA universally promotes <130/80 mmHg as a treatment goal [32]. Meta-analyses support the use of more intensive antihypertensive regimens to reach these targets. Pooled data from 42 randomized trials revealed that achieving SBP between 120-124 mmHg yielded a hazard ratio for CVD events of 0.71 (95% CI: 0.60-0.83) compared to 0.73 (95% CI: 0.58-0.93) for SBP 130-134 mmHg, favoring tighter control [30]. Despite this, more aggressive BP control is not universally advantageous. Evidence from the ACCORD and SPRINT trials shows that while tighter control can reduce cardiovascular risk, it may also increase adverse events, particularly in older adults or those with impaired coronary perfusion. Excessively low diastolic pressure (<60 mmHg) has been linked to increased risks of syncope, renal dysfunction, and even myocardial ischemia. Therefore, individualized treatment goals based on patient comorbidities, functional status, and cardiovascular risk remain essential [33, 34]. Therefore, clinical practice must integrate patient-centered strategies-applying guideline recommendations while tailoring treatment goals to individual circumstances. Shared decision-making is fundamental to improving adherence and minimizing complications [30]. For older patients or those with several health issues, research shows that aiming for moderate BP targets (like 140-150 mmHg) can be helpful without the dangers of trying to lower it too much [35-37].

Chronic hyperglycemia induces endothelial dysfunction, arterial stiffness, and vascular remodeling through oxidative stress and advanced glycation end‑products, exacerbating both macro‑ and microvascular damage in HTN [4, 12]. Risk stratification tools (e.g., atherosclerotic cardiovascular disease (ASCVD) score incorporating age, lipids, BP, and shared‑decision frameworks such as American Diabetes Association and American College of Cardiology joint recommendations on primary prevention of ASCVD in individuals with type 2 diabetes (ADA/ACC joint algorithms)) facilitate tailoring SBP/DBP goals to individual comorbidity profiles, life expectancy, and patient preferences [34]. Socioeconomic challenges, such as low income, limited health literacy, and barriers to care, independently predict poor BP control and medication adherence in HTN. Nonetheless, implementing optimal BP management in the real world presents challenges. Socioeconomic disparities, access to care, and limited patient education often hinder adherence and follow-up. Clinical inertia, inconsistent risk assessment, and variability in physician practice further complicate guideline implementation [12, 25, 30, 38-40]. Moreover, patients with diabetes, particularly those from underserved communities, often face added barriers due to structural health inequities.

Diabetes itself creates a pro-hypertensive vascular environment. Chronic hyperglycemia induces endothelial dysfunction and arterial stiffness through persistent inflammation and oxidative stress [4, 7, 30]. Reactive oxygen species generated under hyperglycemic conditions contribute to vascular remodeling and impaired vasodilation. These pathophysiological changes elevate BP and increase cardiovascular susceptibility, illustrating the importance of effective and individualized BP control in this group. In addition to cardiovascular protection, optimal BP control is essential for preserving renal function in patients with diabetes and CKD. Uncontrolled HTN accelerates glomerular damage and proteinuria, leading to progressive renal dysfunction. Evidence supports targeting systolic BP <130 mmHg in patients with diabetic nephropathy to slow renal decline.

Managing BP after a stroke requires careful timing and strategy. In the early stage after a stroke, high BP can help keep blood flowing to damaged brain tissue. However, persistent HTN can exacerbate cerebral edema or increase hemorrhagic risk, especially in hemorrhagic strokes [14]. Elevated post-stroke BP has also been associated with poor functional outcomes and increased recurrence risk [12]. Further, studies show that rapid or excessive BP lowering in the early hours after ischemic stroke can worsen cerebral hypoperfusion and infarct size [13]. Conversely, delayed control increases the risk of secondary complications. A big study by the BP-lowering treatment Trialists’ Collaboration highlighted that the results of a stroke depend a lot on when treatment is given, the type of stroke, and the initial blood flow to the brain. A cautious approach recommends initiating mild to moderate BP lowering within 24-48 hours post-stroke, particularly once stable cerebral perfusion is confirmed [15].

Long-term, maintaining BP control is critical to prevent recurrent strokes. This includes selecting appropriate antihypertensive classes, optimizing glycemic and lipid control, and reinforcing lifestyle interventions [4, 11]. Clinical guidelines recommend gradual titration to individualized BP goals with close monitoring to prevent iatrogenic hypotension. Ultimately, stroke prevention strategies must consider the patient’s clinical status, stroke characteristics, and the interplay of comorbidities such as diabetes, hypertension, and CKD. Consistent with this, the ACC/AHA and ADA endorse a BP target of <130/80 mmHg for high-risk individuals, including those with diabetes. Meanwhile, ESC/ESH guidelines recommend <140/90 mmHg but allow more aggressive targets based on clinical judgment [28, 32]. Despite variations in numerical thresholds, all major guidelines agree on the importance of personalized, risk-based care planning that considers patient age, comorbidity burden, and tolerance to treatment [25].

Limitations 

It is noteworthy that this narrative review’s non-systematic design might introduce selection bias, given that the included studies were selected based on pertinence to the research questions, publication in reputable peer-reviewed journals, and accessibility via databases, including Web of Science, PubMed, and Google Scholar. For this review, we did not apply any formal search strategy or inclusion/exclusion criteria, and this might have led to the omission of other relevant studies. Additionally, the definitions and measurements of BP control significantly varied across studies. The variability was also methodological as it entailed clinic-based, ambulatory, and home BP monitoring, and threshold-based, including the definition of control as <140/90 mmHg versus <130/80 mmHg. Such discrepancies have complicated the direct comparison of outcomes. Additionally, population heterogeneity across the included studies also poses a challenge concerning interpretation. Thus, most of the included participants had different comorbidities, including diabetes and CKD, and were also on divergent intensities or combinations of antihypertensive treatments, with divergent follow-up durations. These differences are likely to influence the cardiovascular risk profiles and the responses to BP targets, thereby limiting the generalizability of the findings to the wider patient population. Such factors underline the requirement for careful interpretation during the extrapolation of results across contexts and subgroups.

## Conclusions

Proper HTN management significantly reduces the risk of stroke and myocardial infarction, particularly in individuals with HTN. Nonetheless, in persons with diabetes, overly aggressive BP targets might lead to overtreatment, manifested through dizziness, adverse drug effects, syncope, and hypotension. This emphasizes the need for caution against overtreatment, which implies the intensification of antihypertensive therapy beyond the set clinically beneficial thresholds. Individualized BP goals, taking into consideration aspects of age, objectively evaluated risks, and comorbidities, including those drawn from clinical scoring systems such as the ASCVD risk calculator or biomarker profiles, are important in the optimization of therapeutic outcomes. Prospective studies should, therefore, focus on the identification of subgroup-specific BP targets and stratification of outcomes based on the comorbidity profiles so as to improve the safety and accuracy of treatment strategies across divergent patient populations.
